# Chronic Diarrhea

**Published:** 1882-02

**Authors:** 


### CHRONIC DIARRHEA.

CHRONIC diarrhea is generally a symptom only of some other disease, and such cases cannot be successfully treated by remedies that act solely on the intestinal canal. Great grief or nervous anxiety is usually attended with this symptom, which is apt to continue as long as the mental trouble exists. Such cases prove the intimate relations and mutual dependence on each other of the various functions of the body. Every function of the system is performed by the aid of its appropriate nerves under the direction of the nerve centre, the brain. If the brain is worn and fatigued by anxiety the entire nervous system is in sympathy with it, and its tone and vigor is impaired. The bowels are then soonest affected, because in addition to the weakened condition of their nerves a feeble stomach is constantly pouring into them the products of imperfect digestion. The remedies in that case would be those that are best calculated to cure the mental trouble, as cheerful society, travel, etc. This is to be seconded by proper food, frequently given, but in moderation.

What is commonly called consumption of the bowels is a far more serious disease, with fewer chances of recovery, particularly when of long continuance and when there is great emaciation. These troubles should not be allowed to arrive at this stage, and it is here that "an ounce of prevention is worth a pound of cure."

There is a form of chronic diarrhea that attends dyspepsia and which disappears when the dyspepsia is cured.

Last summer I was in correspondence with a gentleman who resides about two hundred miles from New York. His symptoms were peculiar. His general health was excellent, but he was troubled with a painless but persistent diarrhea. Finally, in the latter part of August, he came to see me. Any life insurance company would have regarded him as a prize. I have seldom seen a sounder man, and a careful examination revealed nothing that could account for the constant trouble of the bowels. I had spent perhaps an hour investigating the case and was about to give it up when it occurred to me that his trouble might be owing to the quantity of ice water he drank, for he must have consumed more than a quart while in the office. In fact, this proved to be the solution of the trouble, for by reducing the quantity ninety per cent. he recovered in a week.

